# Physical Activity Telecoaching in Post-Surgical NSCLC Patients: A Mixed-Methods Pilot Study Exploring Feasibility, Acceptability and Actual Usage

**DOI:** 10.3390/cancers17172886

**Published:** 2025-09-02

**Authors:** Eva Arents, Sarah Haesevoets, Fien Hermans, Kirsten Quadflieg, Dries Cops, Maarten Criel, David Ruttens, Veerle Surmont, Bihiyga Salhi, Eric Derom, Thierry Troosters, Dieter Stevens, Chris Burtin, Heleen Demeyer

**Affiliations:** 1 Department of Rehabilitation Sciences, Ghent University, 9000 Ghent, Belgium; eva.arents@ugent.be (E.A.); fien.hermans@ugent.be (F.H.); 2 REVAL–Rehabilitation Research Center, Faculty of Rehabilitation Sciences, Hasselt University, 3590 Diepenbeek, Belgium; sarah.haesevoets@uhasselt.be (S.H.); kirsten.quadflieg@pxl.be (K.Q.); dries.cops@uhasselt.be (D.C.); chris.burtin@uhasselt.be (C.B.); 3 BIOMED–Biomedical Research Institute, Hasselt University, 3590 Diepenbeek, Belgium; 4 Department of Rehabilitation Sciences, KU Leuven, 3000 Leuven, Belgium; thierry.troosters@kuleuven.be; 5 Centre of expertise in Care Innovation, Department of PXL-Healthcare, PXL University of Applied Sciences and Arts, 3500 Hasselt, Belgium; 6 Department of Lung diseases, Hospital Oost-Limburg-Faculty of Medicine and Life Sciences, Hasselt University, 3600 Genk, Belgium; maarten.criel@zol.be (M.C.); david.ruttens@zol.be (D.R.); 7 Department of Pulmonary Medicine, Ghent University Hospital, 9000 Ghent, Belgium; veerle.surmont@uzgent.be (V.S.); bihiyga.salhi@ugent.be (B.S.); eric.derom@uzgent.be (E.D.); dieter.stevens@uzgent.be (D.S.)

**Keywords:** non-small cell lung cancer, physical activity, telecoaching, feasibility, acceptability

## Abstract

Background: Patients with early-stage (I–IIIA) resectable non-small cell lung cancer (NSCLC) often experience reduced physical activity (PA) after surgery. PA telecoaching may support a more active lifestyle, but evidence in this population is limited. Objective: To evaluate acceptability, feasibility, safety, and actual usage of an automated and manual PA telecoaching program following surgery for NSCLC. Methods: In this multicenter, single-blind study, patients received either an eight-week automated coaching program (ACP) with a customized smartphone app or a manual coaching program (MCP) with weekly phone calls from a coach. Both groups used an activity tracker, linked to their smartphone, to monitor steps and receive feedback. Primary outcomes included acceptability, feasibility, safety and usage, assessed via questionnaires and interviews. Secondary outcomes included objectively measured PA (accelerometry), functional exercise capacity (six-minute walk distance) and symptoms (dyspnea, fatigue) and quality of life, evaluated via questionnaires. Results: Nineteen patients (12 males; 68 ± 6 years; baseline daily steps 7820 ± 2799) were included. The majority (18/19) found the intervention enjoyable, and a minority (6/19) reported minor smartphone issues. All patients wore the activity tracker consistently. No adverse events occurred. The ACP required significantly less coach contact time compared to the MCP (25 ± 14 vs. 54 ± 15 min, *p* = 0.0003). No other differences in primary outcomes were observed between groups. Changes in secondary outcomes were limited in both groups. Conclusion: PA telecoaching is feasible, well accepted, and safe in patients with NSCLC post-surgery, with excellent activity tracker adherence. The ACP required less coach involvement. However, increasing PA remains challenging, and no conclusions can be made about the effectiveness of telecoaching. Future research should explore longer interventions in larger populations to assess efficacy and long-term outcomes.

## Simple Summary

An active lifestyle is essential for recovery, quality of life, and long-term health. After surgery for lung cancer, many patients struggle to be physically active. This pilot study explored whether telecoaching—using wearable activity trackers—could support patients towards an active lifestyle. Nineteen individuals participated in an eight-week program. One group received daily guidance and feedback through a smartphone app (automated program), while the other group spoke weekly with a coach by phone (manual program). Nearly all participants found the intervention enjoyable and easy to follow, with excellent adherence to wearing the activity tracker, and no safety concerns were reported. The automated program required less coach time while being equally well received as the manual program. However, increases in daily activity and improvements in symptoms were modest, and the effectiveness of such programs remains uncertain. These results demonstrate that telecoaching is feasible, acceptable, and safe in patients recovering from lung cancer surgery and may offer an efficient way to deliver activity coaching. Future research in larger groups is needed to evaluate long-term benefits and to refine strategies that best help patients increase physical activity in their daily lives.

## 1. Introduction

Lung cancer is the second most commonly diagnosed cancer worldwide and the leading cause of cancer-related death [1]. Non-small cell lung cancer (NSCLC) is the most common type of lung cancer, accounting for about 85% of cases [2]. The standard of care for patients with early-stage (I–IIIA) NSCLC is surgical resection with or without (neo-) adjuvant therapies such as chemotherapy and immunotherapy [3,4].

While these treatments significantly enhance survival rates, they also pose considerable challenges to patients’ physical and emotional well-being, often leading to various symptomatic and physical side effects [5,6]. Current physical activity (PA) guidelines for patients with cancer recommend engaging in at least 150–300 min of moderate-intense activity, 75–150 min of vigorous-intense activity, or a combination of these activities per week [7]. However, fewer than 25% of patients with NSCLC meet these targets 2.9 years post-surgery [8]. Nearly half of patients report persistent dyspnea and fatigue two years post-surgery, which are common barriers to performing PA [9,10,11,12].

Regular PA is crucial for overall health, particularly for populations with chronic diseases such as cancer [13,14,15]. PA interventions for patients with lung cancer are a promising solution because being physically active has been linked to numerous benefits, including reduced symptoms of fatigue and depression, enhanced quality of life (QoL), improved exercise capacity, lower blood pressure and improved survival [10,12,16,17,18,19]. Additionally, PA has been associated with reduced cancer mortality and recurrence across various oncology populations [20]. Recent data indicate that increased PA in patients with resected colon cancer is linked to a 28% lower relative risk of disease recurrence, development of a new primary cancer, or death [21]. As the lung cancer survivor population grows, effective rehabilitation strategies are needed to manage exercise capacity, PA, symptoms, and quality of life (QoL) [22].

To date, research on rehabilitation in lung cancer survivors has primarily focused on the effect of supervised exercise interventions to enhance exercise capacity. While such interventions consistently show enhanced exercise capacity [23], they do not necessarily translate into increased PA [24]. PA encompasses the activities that patients perform in everyday life and within their communities, and it is best understood as a behavioral construct [25]. Therefore, this form of activity requires more than prescribing exercise. It necessitates individualized interventions with behavior change strategies [24], yet this remains scarce in this population. Frameworks such as the COM-B model (“Capability, Opportunity & Motivation” for Behavior Change) emphasize that capability, opportunity and motivation must all be addressed to achieve sustainable behavior change [26]. Wearable devices may support these approaches by enabling self-monitoring, pacing, and motivation to enhance PA.

In recent years, mobile health (mHealth) and telecoaching interventions have emerged as scalable and patient-centered strategies to promote PA, particularly among populations with chronic respiratory diseases [27]. PA telecoaching, a digital intervention that promotes PA through tailored feedback, self-monitoring, and goal setting, has demonstrated positive effects on PA in patients with Chronic Obstructive Pulmonary Disease (COPD) [28] and lung transplant recipients [29]. However, telecoaching did not lead to improvements in PA in patients with interstitial lung disease [30]. These programs focus on increasing daily step count. In oncology, walking-based and digitally supported programs have demonstrated similar feasibility, acceptability, and benefits for psychological and functional outcomes [31,32,33,34].

Despite this progress, evidence for PA telecoaching in NSCLC survivors is lacking. Due to the unique symptom burden, age profile, and comorbidities of this population, as well as the variability in the applicability, relevance, and effectiveness of such intervention across clinical populations, it is essential to first determine whether population-specific adaptations are necessary before evaluating their impact.

Therefore, this pilot study aimed to assess the feasibility, acceptability, actual usage, and safety of an eight-week PA telecoaching program using a Fitbit activity tracker in patients with NSCLC three to nine months post-surgery. As a secondary objective, we explored the impact of PA telecoaching on PA, exercise capacity, symptoms, and QoL.

## 2. Methods

### 2.1. Study Design

This multicenter, two-arm, single-blind study was conducted by Hasselt University and Ghent University in collaboration with three hospitals: Jessa Hospital Hasselt, Hospital East-Limburg and University Hospital Ghent. This increases the representativeness of the study sample for the target population of surgically treated NSCLC patients. Ethics approval was obtained from the relevant committees (Medical Ethical Committee of Ghent University and University Hospital Ghent, Jessa Hospital Hasselt, Hospital East-Limburg and Hasselt University) (ONZ-2022-0012 dd. 5 May 2022).

Participants were randomized (1:1) at baseline to either an automated coaching program (ACP) or a manual coaching program (MCP), stratified by center using sealed envelopes. Patients were blinded to group allocation. They were informed they would receive a PA telecoaching intervention involving a wearable device and weekly goals, but the exact format of delivery (via smartphone app or phone calls) was not disclosed.

Data were collected at baseline (T1) and post-intervention (T2, nine weeks later). The 8-week PA telecoaching started one week following T1 and ended at T2. Written informed consent was obtained before data collection.

### 2.2. Participants

Between June 2022 and December 2023, patients with early-stage NSCLC (I–IIIA) who underwent surgical resection with or without (neo-)adjuvant chemotherapy were included. Patients were enrolled three to nine months after surgery or termination of adjuvant chemotherapy. Exclusion criteria included progressive or recurrent lung cancer, other malignancies within the past two years, psychiatric disorders, or active participation in a rehabilitation program. Individuals unable to use a new electronic device (e.g., smartphone or activity tracker), not proficient in Dutch, or with comorbidities preventing participation in a PA intervention were excluded.

### 2.3. Telecoaching Intervention

Participants received 8-week PA telecoaching via either ACP or MCP. Both interventions were based on the same intervention components; only the way of providing them differed [30]. In brief, the ACP included: (1) a motivational one-on-one interview with the coach to discuss motivation, self-efficacy, barriers, and create an action plan; (2) an activity tracker (Fitbit Charge 4) worn daily to track steps and provide feedback; (3) a patient-tailored smartphone application (m-PAC, AppsOnly) linked to the activity tracker, offering automated coaching with activity goals and feedback; (4) coach-initiated contact for specific issues (e.g., no willingness to increase in PA, technical problems); and (5) a one-page brochure on the importance of regular PA. More information has been published elsewhere [30]. The MCP intervention was similar, except participants did not receive the smartphone application. Instead, they could use the Fitbit app to track their steps and were contacted weekly by telephone for progress updates and feedback. The coaches (Eva Arents and Sarah Haesevoets) had a background of MSc in physiotherapy and had experience in pulmonary rehabilitation. Behavior change techniques were implemented in both interventions [26]. These techniques were used during the motivating one-on-one interview and telephone contacts with participants. The frequency of these contacts was weekly in MCP intervention and coach-initiated for specific issues in the ACP, as mentioned above. Consistency in the interventions across both sites was ensured through the standardization of operational procedures and regular collaborative discussions among coaches throughout the study.

### 2.4. Data Collection

#### 2.4.1. Baseline Characteristics

At T1, age, sex, and Body Mass Index (BMI) (kg/m^2^) were recorded. Additionally, data on lung cancer type, disease stage, type of surgery, and adjuvant therapy were extracted from hospital records.

#### 2.4.2. Primary Outcome Measures

The primary outcome measures were assessed at T2. A project-tailored questionnaire and a semi-structured interview were used to evaluate the acceptability, feasibility, actual usage, and safety for the participants. The project-tailored questionnaire was based on the one used by Loeckx et al. to evaluate patient experience with PA telecoaching for patients with COPD [35]. The semi-structured interview consisted of eight open-ended discussion questions and can be found in Figure S1. The coach responsible at each respective center conducted the interviews, which were transcribed.

#### 2.4.3. Acceptability

Acceptability was defined as the extent to which the participants considered the intervention appropriate. It was based on eighteen questions (Table S1). Four questions regarding acceptability were included in the semi-structured interview: “Did you have prior experience with a smartphone and/or activity tracker?”, “What were your expectations about the intervention?”, “What was your overall experience?” and “What helped you the most in coaching you to a higher PA level?”.

#### 2.4.4. Feasibility

The feasibility of the intervention was evaluated based on the perception of the intervention and was based on two questionnaire questions (“How easy was it to use the smartphone application?” and “How easy was it to learn to use the smartphone application?”) and three interview questions (“Did you have prior experience using a smartphone/activity tracker?”, “How did you experience the technical aspects of this intervention?” and “What do you think of the time spent on the intervention?”). The feasibility from the coach’s perspective was measured by the contact time throughout the study period. Contact time with ACP patients was logged in the back office of the m-PAC, and contact time with MCP patients was logged in an Excel file.

#### 2.4.5. Actual Usage

Actual usage was defined as the extent to which participants used the components of the intervention as intended. In the project-tailored questionnaire, participants were asked to reflect on how often they looked at their activity tracker and used the smartphone application (mPAC application for participants in the ACP group and the Fitbit application for participants in the MCP group). Additionally, a 10-point Likert scale was used to assess the perceived usefulness of the intervention components. Perceived usefulness was categorized as high when participants rated the component with a score of 7 or above on the 10-point Likert scale. Actual usage of the activity tracker was also objectively defined based on the number of days that participants wore the activity tracker, with data derived from the device.

#### 2.4.6. Safety

Adverse events were recorded during the intervention, including their nature, severity, timing (start and stop dates), seriousness, outcome, and suspected cause. These were recorded during telephone contacts between the coach and the participant as well as during clinical visits.

### 2.5. Secondary Outcome Measures

The following secondary outcome measures were evaluated at both T1 and T2: (1) Objectively measured PA via the Dynaport MoveMonitor (McRoberts, The Hague, The Netherlands) for 7 consecutive days following T1 and preceding T2 during waking hours. Participants could only remove the device during water-based activities and at night. Valid measurements were defined as at least 4 days with 8 h of wearing time per day [36], and the average daily step count and moving time were extracted for analysis. (2) Functional exercise capacity was assessed using the six-minute walk test (6MWT), following the ERS/ATS guidelines [37]. The best walking distance from two reliable measures was used, and % predicted values were calculated [38]. (3) Dyspnea was assessed using the modified Medical Research Council (mMRC) dyspnea scale for breathlessness (a higher score indicates a higher level of dyspnea [39]. (4) Fatigue was assessed using the Multidimensional Fatigue Inventory (MFI-20), a 20-item self-report tool (a higher score represents a higher level of fatigue) [40]. (5) Quality of life was assessed using the EORTC QLQ-C30-LC13, a questionnaire assessing disease- and treatment-specific symptoms in lung cancer patients (a higher score represents a higher level of symptomatology) [41]. (6) Beliefs toward PA were assessed at T1 using 10-point Likert scales to assess participants’ perceived importance of PA and the confidence and motivation they had to improve their PA (higher score represents higher levels of importance, confidence and motivation).

### 2.6. Statistical Analysis

To evaluate the acceptability, feasibility, actual usage and safety of the interventions, a mixed-methods analysis was conducted, combining quantitative data from the project-tailored questionnaire with qualitative data from participant interviews. Two researchers (Eva Arents and Sarah Haesevoets) analysed the interview data thematically by using the six-step framework proposed by Braun and Clarke [42] (Table S2).

Statistical analyses were performed using IBM SPSS Statistics version 28 (IBM Corp., Armonk, NY, USA). Continuous data were described as means (M) with standard deviation (SD) or medians (m) with interquartile range (Q1;Q3), depending on the data distribution. Data from the project-tailored questionnaire were presented as categorical variables and reported as proportions or percentages. Non-parametric tests (Wilcoxon signed-rank: within-group; Mann–Whitney U and Fisher’s exact test: between-group) were applied due to heterogeneous data and small sample sizes (*n* ≤ 20). A significance level of 0.05 was set for all statistical analyses.

## 3. Results

**Figure 1:**
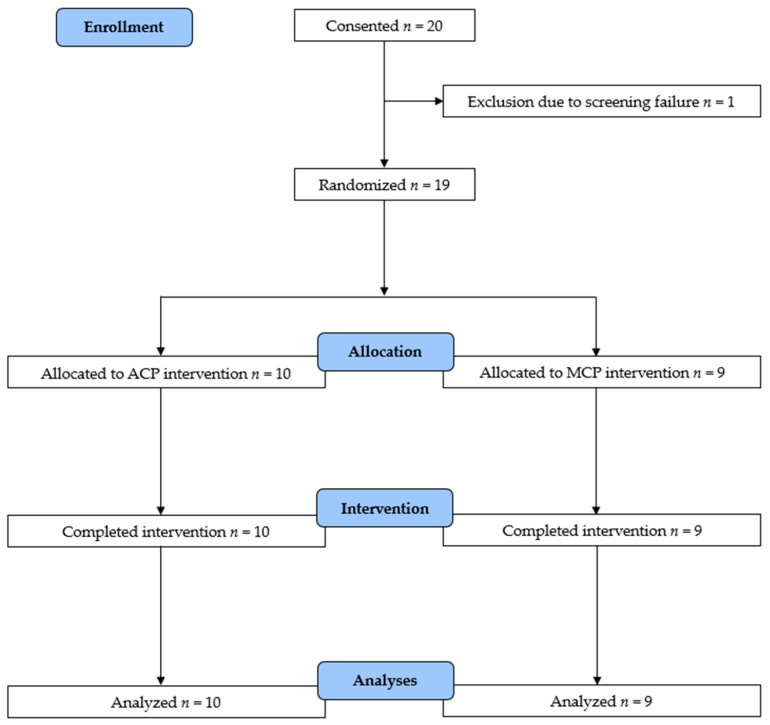
Flow diagram of participants.

Twenty individuals with NSCLC provided consent, and nineteen were randomized into the ACP or MCP group. One patient was excluded due to a screening failure (Figure 1). All participants completed the intervention, and there were no dropouts. A detailed description of the baseline characteristics (*n* = 19) is provided in Table 1. Participants were similar in terms of sex, age, and BMI. The average time between surgery and randomization was 145 ± 50 days for the total patient sample, and this did not differ significantly between groups. Those in the ACP group had significantly higher fatigue levels, as measured by the MFI-20 (56 (48;62) vs. 39 (31;52) points, *p* = 0.04), took fewer daily steps (6696 ± 2656 vs. 9070 ± 2520, *p* = 0.04) and had less moving time (83.2 ± 27.6 vs. 108.4 ± 27.4 min/day, *p* = 0.04) than to those in the MCP group. Patients wore the activity monitors for an average of 843 ± 140 min per day over 6 ± 1 days. Beliefs toward PA were comparable between groups. Two participants in the MCP group and six participants in the ACP group received (neo-)adjuvant chemotherapy (*p* = 0.12). More detailed information is available in Table S3.

### 3.1. Acceptability

#### 3.1.1. Experiences/Expectations Before the Intervention

Most participants (17/19) had prior experience with smartphones, but 14/19 had never used an activity tracker (78% in MCP, 70% in ACP). Before starting the intervention, 8/19 participants had no clear expectations, 4 expected to increase motivation, 3 hoped to improve their overall condition, and 4 had general positive expectations.

#### 3.1.2. Experiences/Feedback at the End of the Intervention

PA telecoaching was well received by patients in both groups as 18/19 participants indicated that they enjoyed the intervention, and 9 reported an increased motivation toward PA during the interview. MCP participants stated: “*It motivated me a lot*” (ID 1); “*I’ll buy an activity tracker myself*” (ID 2); “*It truly worked*” (ID 15). ACP participants said: “*It got me off my sofa*” (ID 3); “*The activity tracker motivated me to walk more*” (ID 6); “*It encouraged me to go outside*” (ID 12). All patients experienced the weekly step goal as “good”, except for two patients in the ACP group who indicated that they experienced the step goal as “slightly too high”. However, one patient in the MCP group did mention that not reaching the weekly goal sometimes demotivated him. He stated: *“It demotivated me when I saw that my number of steps were below the step goal, I even removed my activity tracker for a while”*(ID4). One patient in the ACP group (ID3) considered the customized app to be rather basic and expressed interest in including additional health parameters such as heart rate, blood pressure, and oxygen saturation to enhance its usefulness.

#### 3.1.3. Impact of Different Telecoaching Components

Participants in both groups reported high satisfaction with the activity tracker and the feedback it provided. Those in the MCP group also rated the weekly phone calls positively. There were no significant differences in satisfaction between the groups. The ACP-specific components (daily activity goal, feedback, performance graphs, and weekly tips) received high ratings from this group (median score = 7.5–9.5/10) (Table 2).

**Figure 2:**
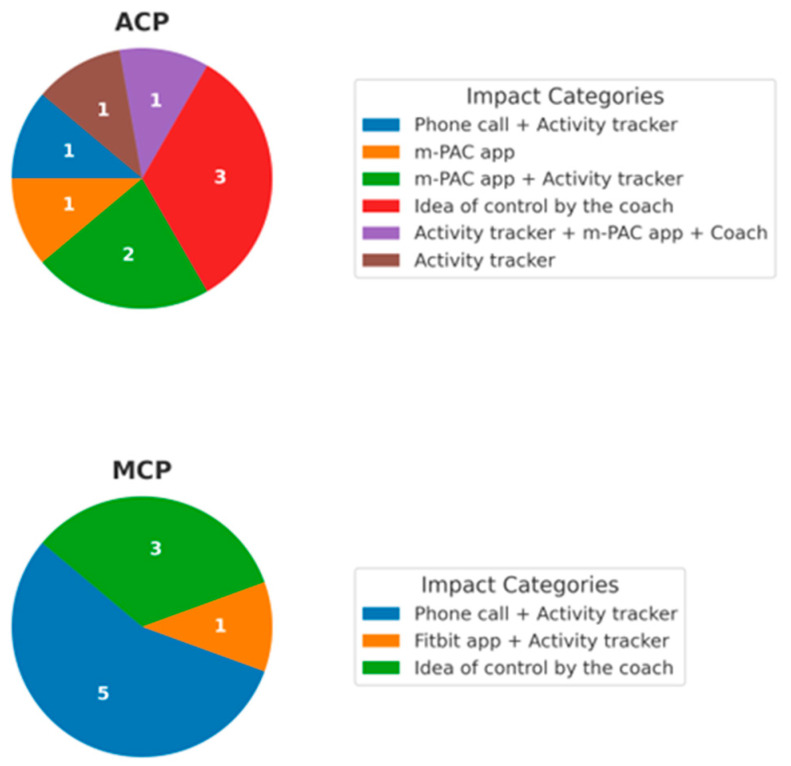
Pie charts show components patients rated as having the “highest impact on activity” in interviews. Information about one ACP patient is missing.

During the interview, 6/9 (information for one patient is missing) in the ACP group and 5/9 in the MCP group indicated that the activity tracker had the greatest impact on improving their PA level, especially when combined with phone calls (see Figure 2).

In the project-tailored questionnaire, both groups rated the smartphone application, either the customized app in the ACP group or the Fitbit app in the MCP group, with at least 3/5 stars. When asked about their willingness to use the application again in the following year, only one participant in each group indicated that they did not want to use it further at all. However, only one participant in the MCP group and none in the ACP group said they would pay for the application if it were a paid service. Further details on acceptability are provided in Table S3.

### 3.2. Actual Usage

In the project-tailored questionnaire, 9/10 participants in the ACP group reported checking the activity tracker multiple times a day, compared to 7/9 in the MCP group. 8/10 (ACP) and 9/9 (MCP) of participants looked at the application at least once daily (Table S4). Compliance with wearing the activity tracker was high in both groups (MCP: 7 (7;7) days/week vs. ACP: 7 (6.9;7) days/week), and there were no significant differences between the groups (*p* = 0.50) (Table S4).

### 3.3. Feasibility

4/9 (MCP) and 5/10 (ACP) indicated that they could immediately use the applications (Fitbit for MCP and m-PAC for ACP). Over time, 4/9 (MCP) found it manageable to become familiar with the apps. Most participants rated the apps as easy to use (7/9 MCP, 4/10 ACP), while 5/10 (ACP) found them “very easy”. A small number of participants (3/9 in MCP and 3/10 in ACP) reported minor technical issues with the activity tracker during the semi-structured interviews, such as difficulties with the screen contrast, charging, and synchronizing step data to the application. These issues were resolved with coach support. One ACP participant (ID3), who had no prior smartphone experience, received clear instructions. He later emphasized that the smartphone application was the most important part of the intervention and stating: “*I had some start-up problems, but after contacting you, these were solved*.”

During the 8 week intervention, the ACP group had significantly less contact time with the coach than the MCP group (25 ± 14 vs. 54 ± 15 min, *p* = 0.003). Of all phone calls in the ACP group, 61% were coaching-related, 20% were made to resolve technical issues, and 19% were for other reasons. Of the coaching-related calls, 32% were to inform patients about the first step goal in the customized smartphone app, and 48% involved discussing with patients why their step goal had not been increased in the past two weeks. The remaining 20% addressed the absence of the weekly Sunday evening report for two consecutive weeks. Participants in the MCP were contacted weekly by phone by the coach, and 100% of these calls were coaching-related. Participants in both groups emphasized the importance of the interaction with the coach. One participant stated “*The feeling that you could follow my performance really motivated me to increase my physical activity*” (ID9). Detailed feasibility information for both groups is provided in Table S5. MCP patients increased their step goal more than the ACP patients (43 ± 7% vs. 28 ± 14% of the time, *p* = 0.008). MCP patients decreased their step goal more often than the ACP patients did (14 ± 9 vs. 4 ± 4 % of the time, 0.010), and more ACP patients maintained the same step goal as the previous week than the MCP patients did (68 ± 15 vs. 43 ± 9 % of the time, *p* = 0.004).

### 3.4. Safety

No adverse events related to the intervention were reported by participants to the coach during the intervention period.

### 3.5. Secondary Outcomes

Changes in secondary outcomes are included in Table 3. Overall, there was no change in objectively measured PA levels. A significant improvement in 6MWD (+15 ± 42 m, *p* = 0.03) and a trend towards less lung cancer-specific symptoms (−3 (−6;0) points, *p* = 0.05) was observed. A significant decrease in fatigue (−14 (−17;−8) points, *p* = 0.005) and significant improvement in daily step count (+759 ± 933 steps/day, *p* = 0.04) were observed in the ACP group. A trend towards significant improvement in 6MWD (+28 ± 37 m, *p* = 0.05) was observed in the MCP group. No significant within-group differences were observed, except for significantly lower fatigue in the ACP group compared to the MCP group (*p* < 0.001).

## 4. Discussion

This pilot study examined the acceptability, feasibility, actual usage, and safety of a PA telecoaching in patients with NSCLC following surgical resection. PA telecoaching was provided for 8 consecutive weeks by either an ACP or an MCP and included an activity tracker (Fitbit Charge 4) to track daily step count and provide feedback. The findings indicate that both interventions were well-accepted with high activity tracker adherence and positive feedback from participants. Additionally, both interventions were safe, with no adverse events reported. However, patients were generally not prepared to pay for this service.

### 4.1. Comparison of Findings to Previous Research

Feedback from questionnaires and interviews indicates that participants enjoyed the free intervention. These findings align with previous research in patients with COPD and lung transplant recipients [29,35]. As expected, the ACP intervention required significantly less coaching time, suggesting greater efficiency but, importantly, without reducing engagement. Both groups valued the coach supervision, particularly the phone support, which highlights its motivational role. These findings are supported by Hume et al. (2022) [29] who tested a similar intervention and found that nearly half of the patients after lung transplantation rated their interactions with the coach as highly important. Additionally, patients in the MCP received more contact from coaches, and these patients increased their step goal more often as compared to patients in the ACP group. Participants in both intervention groups did not find the intervention burdensome. Even those without prior smartphone experience adapted well, reinforcing its accessibility for both persons with and without smartphone experience. Engagement was high, with most patients regularly interacting with the activity tracker and smartphone application.

These findings are comparable to those from the study of Vorrink et al. (2016) [43], who also reported high activity tracker adherence (median days worn per week = 6.68/7) in patients with COPD during a six-month coaching intervention following pulmonary rehabilitation. Similar to our results, they found no significant change in physical activity levels after the coaching intervention. However, participants rated activity tracker as highly useful, suggesting its value in supporting autonomy and motivation. This is further supported by Donnachie et al. [44], who found that activity tracker use enhanced participants’ sense of autonomy to increase physical activity in a population of overweight and obese men.

Both telecoaching interventions in the present study align with the COM-B system (“Capability, Opportunity & Motivation” for Behaviour change) [45] where ‘capability’ was addressed as the included patients were treated with curative upset and presented with preserved functional exercise capacity. ‘Opportunity’ (to become more physically active) was addressed during the one-on-one interview at T1 where goals, barriers and facilitators were discussed. This was further supported by the regular phone calls in the MCP group and the phone calls for specific issues in the ACP group. ‘Motivation’ was targeted through direct feedback from the activity tracker and telephone calls in both groups as well as the automatic messages that ACP patients received on their smartphones (daily display of activity goals, daily and weekly feedback, cumulative achievements and positive tips to stay active). These messages were well-received.

Effectiveness of the interventions (secondary outcomes) was lower than expected. However, interpretation of these findings is complicated by baseline imbalances between the ACP and MCP groups. Notably, participants in the ACP group started with significantly lower step counts, less moving time, and higher fatigue scores. These differences may have acted as confounding factors, limiting the comparability of post-intervention outcomes. Furthermore, the improvements observed in step count and fatigue within the ACP group–and in 6MWD in the MCP group–could partially be attributed to regression to the mean rather than a true intervention effect. Regression to the mean is particularly likely in small pilot studies where extreme baseline values are more common due to random variation and cannot be fully corrected by randomization alone [46]. Therefore, the current findings do not allow firm conclusions regarding the effectiveness of either intervention. Future studies with larger sample sizes could use stratified randomization to account for baseline disparities and minimize bias.

Overall, PA telecoaching did not lead to consistent improvements in secondary outcomes, with exception of a statistically significant, but not clinically, meaningful improvement in 6MWD (+15 ± 42, *p* = 0.03). Longer intervention durations (≥12 weeks) in similar populations (e.g., patients with COPD, lung transplant recipients) [28,29,47] have reported greater improvements in PA (870 to 3475 steps) although other programs have shown no effect [30,43]. This highlights the importance of tailored intervention strategies (e.g., more or less coach supervision needed) and appropriate patient selection (e.g., individuals with lower baseline PA) to maximize potential benefits of telecoaching.

### 4.2. Strengths and Limitations

The strengths of this study include the application of evidence-based behavior change techniques to enhance motivation for PA [26], and a comprehensive evaluation of the feasibility and acceptability two different telecoaching interventions through both quantitative and qualitative methods. Braun and Clarke’s six-step framework [42] was used for interview analysis to ensure consistency. A COPD-based questionnaire and application previously used were employed to facilitate comparison with previous studies [35,47].

As the intervention was designed to be accessible for individuals with diverse levels of digital literacy (coaches provided smartphones to patients who did not own one), adherence was high, and there were no dropouts. Lastly, feasibility was assessed from the perspective of both the participants and coaches, providing a comprehensive understanding of the intervention’s burden.

However, this study has some limitations that should be considered when interpreting the results. First, as a pilot study with a small sample size, it was designed to assess feasibility, acceptability, actual usage and safety of an intervention; therefore, its ability to draw conclusions regarding effectiveness is limited. Furthermore, participants were already fairly active at baseline, which may have affected the low impact of the interventions on secondary outcomes. Additionally, no data were collected on comorbidities or lung function, which may have also have influenced the limited impact observed. Second, previous research shows that coaches’ skills improve with more coaching experience [35]. In our study, the small sample size may have constrained the coaches’ learning curve, thus reducing their effectiveness on secondary outcomes. Third, the predominantly male population (89%) may restrict the generalizability of the results to female patients. Fourth, selection and answering biases may have occurred, as motivated patients may have been more inclined to participate. Motivation was only assessed at baseline, making it impossible to analyze changes over the intervention period. Finally, coaching variability across centers may have introduced inconsistencies, though adherence to behavior change techniques and regular meetings helped mitigate this concern.

### 4.3. Clinical Importance and Future Recommendations

The integration of digital technologies into healthcare is becoming more common, providing new opportunities for innovation and improvement [27]. With the rise in smartphone use [48], digital health applications such as app-based PA telecoaching offer a significant advantage by enabling remote guidance, thereby reducing the burden on both healthcare providers and patients. This approach makes PA programs more convenient, time-efficient, and cost-effective, as patients can participate from home or while traveling. As demonstrated by the present study, using an automated approach decreases coaches’ time spent guiding patients, thereby increasing overall efficiency without negatively influencing the patient’s experience in the short term. However, digital interventions may not suit all patients, particularly those without access to smartphones or activity trackers or those who prefer face-to-face interactions with coaches. Similar studies show that combining activity trackers with direct coach contact boosts PA levels [29,47]. This suggests that digital programs should be tailored to individual needs, with coaches selecting the most appropriate approach. Additionally, such programs should be implemented at low or no cost to patients to ensure adoption.

This pilot study demonstrates that PA telecoaching is feasible, acceptable, and safe for patients with NSCLC recovering from surgical resection. The high adherence and positive participant feedback indicate the potential for integrating this approach into post-operative care. However, the effect of the interventions should be further studied in properly powered studies, as it can be questioned. Based on the present preliminary data, the short-term benefits of objectively assessed physical activity seem to be limited to ACP. Future research should extend the intervention duration to enhance effectiveness, broaden patient inclusion criteria to target individuals earlier post-surgery, identify those most likely to benefit (e.g., patients with lower baseline activity levels), incorporate alternative PA strategies to overcome environmental barriers, enable intervention customization, and assess long-term adherence, PA, exercise capacity, symptoms, and QoL outcomes through extended follow-up.

## 5. Conclusions

This pilot study demonstrates that physical activity telecoaching is feasible and well accepted among patients with NSCLC following surgical resection. High adherence rates, frequent usage of the activity tracker and app, and positive participant feedback reflect strong user engagement. Notably, the semi-automated coaching format required less coach involvement, indicating potential for efficient implementation. However, given the small sample size and baseline group imbalance, no conclusions can be drawn regarding the effectiveness of either intervention. A well-powered randomized controlled trial is needed to determine the efficacy, long-term outcomes, and optimal intervention strategies for increasing physical activity in this population.

## Figures and Tables

**Table 1:** Baseline characteristics of participants.

Variable	All Participants	MCP Group *n* = 9	ACP Group *n* = 10	*p*-Value
Sex (male), *n* (%)	12 (63)	5 (55)	7 (70)	0.43
Age (years)	68 ± 6	69 ± 7	66 ± 5	0.18
BMI (kg/m^2^)	25 ± 5	24 ± 3	26 ± 6	0.45
Timing since surgery (days)	145 ± 50	165 ± 58	127 ± 35	0.21
6MWD (m)	548 ± 72	513 ± 51	579 ± 76	0.05
6MWD (% pred)	95 ± 11	91 ± 8	98 ± 13	0.16
MFI-20 (20–100)	50 (33;57)	39 (31;52)	56 (48;62)	0.04
HADS Anxiety score (0–21)	5.0 (3.0;8.25)	5.0 (0.75;6.75)	6.0 (3.75;9.25)	0.32
HADS Depression score (0–21)	3.5 (1;7)	3.5 (1.5;8.0)	4.0 (1.0;7.0)	0.97
mMRC score (0–4)	1 (1;2)	1 (1;2)	1 (0;1.25)	0.24
EORTC-QLQ-LC13 score (0–100)	13.9 (8.3;25)	11.1 (6.9;25.0)	19.4 (11.8;23.6)	0.45
Step count (steps/day)	7820 ± 2799	9070 ± 2520	6696 ± 2656	0.04
Moving time (min/day)	95.2 ± 29.7	108.4 ± 27.4	83.2 ± 27.6	0.04
Importance PA (0–10)	8.0 (8.0;10.0)	9.0 (8.0;10.0)	8.0 (7.0;9.3)	0.24
Motivation PA (0–10)	8.0 (8.0;9.0)	8.0 (8.0;8.5)	8.0 (7.8;9.0)	0.78
Confidence PA (0–10)	7.0 (7.0;8.0)	7.0 (6.0;8.0)	7.0 (7.0;8.3)	0.66
Season of intervention start				
Spring, n (%)	3 (16)	1 (11)	2 (20)	0.80
Summer, n (%)	7 (37)	4 (44)	3 (30)	
Autumn, n (%)	5 (26)	3 (33)	2 (20)	
Winter, n (%)	4 (21)	1 (11)	3 (30)	

Note: Data are reported as mean ± SD or median (Q1;Q3), unless specified differently. ACP: Automated Coaching Program; MCP: Manual Coaching Program; *n*: sample size; BMI: Body Mass Index; Timing since surgery = number of days between randomization and surgery date; 6MWT: Six-Minute Walking Distance; pred: predicted; MFI-20: Multidimensional Fatigue Inventory; HADS: Hospital Anxiety and Depression Scale; mMRC: modified Medical Research Council scale; EORTC- QLQ-LC13: European Organization for the Research and Treatment of Cancer Quality of Life Questionnaire and Lung Cancer Module; PA: physical activity. Mann–Whitney U and chi-square tests were performed.

**Table 2:** Perceived usefulness of Intervention Components.

Component	MCP Group	ACP Group	*p*-Value
Wearing activity tracker	10 (9;10)	10 (8;10)	>0.99
Feedback activity tracker	9 (8;10)	10 (9;10)	0.40
Phone calls with coach	9 (9;10)	10 (9;10)	0.39
Daily activity goal		9 (7;10)	
Daily feedback		9 (6;10)	
Performance graphs		10 (8;10)	
Tip of the week		7.5 (6;9)	

Note: Data are reported as median (Q1;Q3). Daily activity goal, daily feedback, performance graphs and tip of the week messages were specific to the ACP intervention. Components were rated using a 10-point Likert scale. ACP: Automated Coaching Program; MCP: Manual Coaching Program. Mann–Whitney U tests were performed.

**Table 3:** Changes in physical activity, functional exercise capacity, and symptoms.

	Overall		MCP Group		ACP Group		
Variable	Within-Group Difference	*p*-Value	Δ	*p*-Value	Δ	*p*-Value	*p*-Value (Between-Group Δ)
Step count (steps/day)	−253 ± 2426	0.33	−1266 ± 3054	0.44	759 ± 933	0.04	0.08
Moving time (min/day)	−1.7 ± 26.6	0.33	−12.9 ± 33.3	0.52	9.4 ± 11.0	0.05	0.06
6MWD (m)	15 ± 42	0.03	29 ± 37	0.05	3 ± 44	0.31	0.18
MFI-20	−6 (−14;0)	0.10	0 (−6;18)	0.40	−14 (−17;−8)	0.005	< 0.001
HADS Anxiety score	1 (−1;2)	0.39	1 (−2;3)	0.57	1 (−1;3)	0.57	0.90
HADS Depression score	−1 (−2;1)	0.36	−1 (−3;0)	0.18	−1 (−2;2)	0.91	0.52
mMRC score	0 (−1;0)	0.19	0 (−1;0)	0.18	0 (−1;0)	0.48	0.84
EORTC-QLQ-LC13 score	−3 (−6;0)	0.05	−3 (−3;1)	0.33	−4 (−9;1)	0.09	0.21

Note. Data are reported in mean ± SD or median (IQ1;IQ3). ACP: Automated Coaching Program; MCP: Manual Coaching Program; M: Mean; SD: Standard Deviation; 6MWD: Six-Minute Walking Distance; MFI-20: Multidimensional Fatigue Inventory; HADS: Hospital Anxiety and Depression Scale; mMRC: modified Medical Research Council scale; EORTC-QLQ-LC13: European Organization for the Research and Treatment of Cancer Quality of Life Questionnaire and Lung Cancer Module. Δ: T2-T1. Wilcoxon Rank tests for within-group and Mann–Whitney U and chi-square tests for between-group were performed.

## Data Availability

The data presented in this study are available upon request from the corresponding author.

## Supplementary Materials

The following supporting information can be downloaded at: https://www.mdpi.com/article/10.3390/cancers17172886/s1, Figure S1: Discussion guide; Table S1: Acceptability of the ACP and MCP interventions (project-tailored questionnaire); Table S2: Six-step Thematic Framework as proposed by Braun and Clarkes (2006); Table S3: General Participant Information; Table S4: Actual Usage of the ACP and MCP Interventions; Table S5: Feasibility of the ACP and MCP Intervention.
