# Supplementary material for: Physical Activity Telecoaching in Post-Surgical NSCLC Patients: A Mixed-Methods Pilot Study Exploring Feasibility, Acceptability and Actual Usage
Source: Cancers (Basel). 2025 Sep 2;17(17):2886. doi: 10.3390/cancers17172886 (PMC12427458; doi:10.3390/cancers17172886)
Supplement: Supplementary file 1 [file cancers-17-02886-s001.zip › cancers-3778345-supplementary.pdf]

Supplementary Material

Figure S1

Discussion guide

1) Did you have prior experience using a smartphone? Did you have prior experience using an activity tracker?

2) What were your expectations of the intervention?

3) What was your overall experience with the intervention?

4) How did you experience the technical aspects of the intervention? What difficulties did you encounter? What was easy for you? (Please discuss the mobile phone, the pedometer, user- friendliness, etc.)

5) What helped or supported you the most in coaching towards a higher level of physical activity?

6) What did you find to be the positive and negative aspects of this coaching intervention?

7) What is your opinion on the amount of time you spent on the intervention?

8) Do you have any other comments about the coaching program that you would like to share? Any suggestions?

Table S1. Acceptability of the ACP and MCP interventions (project-tailored questionnaire).

Project tailored questionnaire	Answers	MCP N (%)	ACP N (%)	P-value
Questions regarding the telecoaching smartphone application (ACP group) and Fitbit application (MCP group)				
1) Is the application enjoyable/entertaining to use?	Totally not enjoyable or entertaining	0 (0)	1 (10)	>0.99
	Mostly boring	0 (0)	0 (0)	
	Ok, enjoyable enough to be entertained for a short period (<5min)	2 (22)	3 (30)	
	Rather enjoyable and entertaining for an average period of 5-10 min	1 (11)	1 (10)	
	Very enjoyable and entertaining, stimulating to use multiple times	6 (76)	5 (50)	
2) Is the application interesting to use? Does it present itself in an interesting way?	Totally not interesting	0 (0)	0 (0)	0.70
	Mostly not interesting	0 (0)	1 (10)	
	Ok, stimulating to use the application for a shorter period (<5min)	0 (0)	1 (10)	
	Fairly interesting, stimulating to use the application for an average period of 5-10min	4 (44)	2 (20)	
	Very interesting, stimulating to use multiple times	5 (56)	6 (60)	
3) How accurate/fast do all functions in the application and the different components (buttons/menu) work?	App does not work	0 (0)	0 (0)	0.96
	Some functions do work, but not optimal or have large technical problems	0 (0)	0 (0)	
	App works overall well but some technical problems need to be fixed/app is sometimes slow	2 (22)	2 (20)	
	App mostly functions well, some minor problems	4 (44)	4 (40)	
	Perfect/timeable answers, no technical problems	3 (33)	4 (40)	
4) Is changing screens logic/accurate; are all buttons that are needed to change screens present?	Multiple components are placed randomly, the overview is confusing, navigating the app is difficult.	0 (0)	0 (0)	>0.99
	Manageable after spending a lot of time	1 (11)	0 (0)	
	Manageable after spending some time	3 (33)	3 (30)	
	Easy to use, some links to change screens are missing	2 (22)	2 (20)	
	Perfectly logic, clear and easy to change screens	3 (33)	5 (50)	
5) Are the interactions consistent and clear throughout all screens and components of the application?	Completely inconsistent/confusing	0 (0)	0 (0)	>0.99
	Often inconsistent/confusing	0 (0)	0 (0)	
	OK, with a few inconsistent/confusing elements	1 (11)	1 (10)	
	Mostly consistent/clear with minor problems	5 (56)	6 (60)	
	Perfectly consistent and clear	3 (33)	3 (30)	
6) Is the arrangement and size of the buttons/icons/menus on the screen appropriate, or can you adjust the size if you want?	Very poor design, cluttered, some items/options are impossible to select/see/read/find, screen display not optimized	0 (0)	0 (0)	0.76
	Poor design, random, unclear, some items/options are difficult to select/see/read/find	1 (11)	0 (0)	
	Satisfactory, some issues with selecting/seeing/reading/finding items/options or minor screen size problems	1 (11)	3 (30)	
	Mostly clear, it is possible to select/see/read/find items/options	4 (44)	3 (30)	
	Professional, simple, clear, organized, logically structured, screen display is optimized, every design element has a purpose	3 (33)	4 (40)	
7) How high is the quality/resolution of the graphics used for the buttons/icons/menus in the application?	The graphics look amateurish, very poor visual design, inconsistent style	0 (0)	0 (0)	0.71
	Low quality/resolution graphics, poor visual design, inconsistent style	0 (0)	0 (0)	
	Average quality graphics and visual design (generally consistent style)	3 (33)	3 (30)	
	High quality/resolution graphics and visual design, consistent style.	5 (56)	4 (40)	
	Very high quality/resolution graphics and visual design, consistent style throughout the entire app	1 (11)	3 (30)	
8) How good does the app look?	No visual appeal, unpleasant to look at, poor design, mismatched colors	0 (0)	0 (0)	0.31

	Little visual appeal, poor design, incorrect use of colors, visually dull	0 (0)	0 (0)	
	Some visual appeal, average, neither exciting nor dull	2 (22)	4 (40)	
	Highly visually appealing, seamless graphics, consistent and professional design	5 (56)	2 (20)	
	As above, plus very attractive, stands out, use of colors enhances app functions/menu navigation	2 (22)	4 (40)	
9) Is the content of the app correct, well-written, and relevant to the app's topic? Does the app provide enough information?	Not applicable: No information is provided in the app	0 (0)	0 (0)	0.50
	Irrelevant/inappropriate/incoherent/incorrect	0 (0)	0 (0)	
	Slightly relevant/appropriate/coherent/sometimes incorrect	0 (0)	0 (0)	
	Moderately relevant/appropriate/coherent/often correct	0 (0)	2 (20)	
	Relevant/appropriate/coherent/correct	4 (44)	5 (50)	
	Highly relevant/appropriate/coherent and correct	5 (56)	3 (30)	
10) Is the visual explanation of concepts clear, logical, and correct – through graphs/images/videos?	Not applicable: No visual information is used in the app (e.g., text only)	0 (0)	0 (0)	0.65
	Very unclear/confusing/incorrect or missing necessary visual explanations	0 (0)	0 (0)	
	Mostly unclear/confusing/incorrect	0 (0)	0 (0)	
	OK, but often unclear/confusing/incorrect	0 (0)	0 (0)	
	Mostly clear/logical/correct, with negligible issues	6 (67)	5 (50)	
	Perfectly clear/logical/correct	3 (33)	5 (50)	
11) Would you recommend this app to people who might benefit from it?	Not at all – I wouldn’t recommend this app to anyone	0 (0)	0 (0)	>0.99
	There are very few people I would recommend this app to	0 (0)	1 (10)	
	Maybe – there are some people I would recommend it to	1 (11)	1 (10)	
	There are many people I would recommend this to	4 (44)	4 (40)	
	Definitely – I would recommend this app to everyone	4 (44)	4 (40)	
12) How often do you think you will use this app in the next 12 months?	Not at all	1 (11)	1 (10)	0.88
	1-2 times	1 (11)	2 (20)	
	3-10 times	2 (22)	1 (10)	
	11-50 times	2 (22)	1 (10)	
	More than 50 times	3 (33)	5 (50)	
13) Would you pay for this app?	No	2 (22)	6 (60)	0.17
	Maybe	6 (67)	4 (40)	
	Yes	1 (11)	0 (0)	
14) What is your overall rating of this app?	★ One of the worst apps I’ve ever used	0 (0)	0 (0)	>0.99
	★★	0 (0)	0 (0)	
	★★★ Average	2 (22)	3 (30)	
	★★★★	6 (67)	5 (50)	
	★★★★ One of the best apps I’ve ever used	1 (11)	2 (20)	
Questions regarding overall telecoaching experience				
1) How much did you enjoy participating in this coaching program?	I enjoyed it very much	3 (33)	3 (30)	>0.99
	I enjoyed it	6 (67)	6 (60)	
	Neutral	0 (0)	1 (10)	
	I did not enjoy it	0 (0)	0 (0)	

	No opinion	0 (0)	0 (0)	
2) Did the intervention help coach you in increasing your physical activity?	Yes, it helped me a lot	7 (78)	8 (80)	>0.99
	Yes, it helped me a little	2 (22)	2 (20)	
	No, it rather discouraged me	0 (0)	0 (0)	
	No, it strongly discouraged me	0 (0)	0 (0)	
3) How did you experience the weekly step goal increase during the intervention?	Much too low	0 (0)	0 (0)	0.48
	Slightly too low	0 (0)	0 (0)	
	Good	9 (100)	8 (80)	
	Slightly too high	0 (0)	2 (20)	
	Much too high	0 (0)	0 (0)	
4) What was the most important component of the telecoaching intervention for you?	Activity tracker	7 (78)	6 (60)	0.78
	Application	1 (11)	3 (30)	
	Activity brochure	0 (0)	0 (0)	
	Telephone contact with the coach	1 (11)	1 (10)	
	Other	0 (0)	0 (0)	
Data Interview				
Questions	Technical aspect	Explanation	Explanation	
1) Did uou have experience using a smartphone and activity tracker?	Smartphone	Yes: 8 (89)	Yes: 9 (90)	>0.99
	Activity tracker	Yes: 2 (22)	Yes: 4 (40)	>0.99

Note. ACP: Automated Coaching Program; MCP: Manual coaching Program; Min: minutes. Fisher's exact tests were performed.

Table S2. Six-step Thematic Framework as proposed by Braun and Clarkes (2006).

Phase	Description of the phase
1. Familiarizing yourself withyour data	Transcribing data (if necessary), reading and re-reading thedata, noting down initial ideas
2. Generating initial codes	Coding interesting features of the data in a systematic fashionacross the entire data set, collating data relevant to each code
3. Searching for themes	Collating codes into potential themes, gathering all datarelevant to each potential theme
4. Reviewing themes	Checking if the themes work in relation to the coded extracts (Level 1) and the entire data set (Level 2), generating a thematic 'map' of the analysis
5. Defining and naming themes	Ongoing analysis to refine the specifics of each theme, and the overall story the analysis tells, generating clear definitions and names for each theme
6. Producing the report	The final opportunity for analysis. Selection of vivid, compelling extract examples, final analysis of selectedextracts, relating of the analysis to the research question and literature, producing a scholarly report of the analyses.

Table S3. General Participant Information.

Study ID	Sex	Age	Coaching group	Lung cancer type	NSCLC stage	Surgery type	Adjuvant therapy	Timing since surgery (days)
1	Male	64	MCP	Adenocarcinoma	IA2	VATS Lobectomy left upper lobe	/	234
3	Male	70	ACP	Adenocarcinoma	IIIB	Bilobectomy right upper and middle lobe	Neoadjuvant chemo (carbo-pem)	100
4	Male	76	MCP	Adenocarcinoma	IIIA	VATS Lobectomy right upper lobe	Chemotherapy started during intervention	179
5	Male	60	ACP	Spinocellular	IA2	VATS complete lobectomy right lobe	/	98
6	Male	67	ACP	Low differentiated adenocarcinoma	IIIA	Bilobectomy right upper middle lobe	Chemotherapy (carbo-pem)	160
7	Male	73	MCP	Adenocarcinoma	IIA	RATS Lobectomy light lower lobe	/	84
8	Female	56	MCP	Adenocarcinoma	IB	VATS lobectomy right upper lobe	/	94
9	Female	64	ACP	Adenocarcinoma	IB	VATS lobectomy right upper lobe	/	92
10	Female	62	ACP	Adenocarcinoma	IA3	RATS lobectomy right lower lobe	/	95
11	Male	63	ACP	Squamous cell carcinoma	IIB	VATS lobectomy right lower lobe	Chemotherapy (cis-vino)	119
12	Male	64	ACP	Adenocarcinoma	IIIA	VATS lobectomy right lower lobe	Chemotherapy (gem-cis)	146
13	Female	75	ACP	Adenocarcinoma	IA	VATS lobectomy right lower lobe	/	112
14	Male	75	MCP	Adenocarcinoma	IIA	VATS lobectomy right lower lobe	/	196
15	Female	68	MCP	Adenocarcinoma	IIA	VATs lobectomy left upper lobe	/	112
16	Male	72	ACP	Adenocarcinoma	IB	VATS lobectomy right upper lobe	Chemotherapy	194
17	Male	62	ACP	Squamous cell carcinoma	IIIA	VATS lobectomy left lower lobe	Chemotherapy (carbo-gem)	153
18	Male	73	MCP	Adenocarcinoma	IA	VATS lobectomy right upper lobe	/	219
19	Female	78	MCP	Adenocarcinoma	IIA	VATS lobectomy left upper lobe	/	145
20	Female	62	MCP	Adenocarcinoma	IIB	VATS lobectomy left upper lobe	Chemotherapy (gem-cis)	225

Note: : Type of adjuvant chemotherapy was not specified in the medical records of ID16. NSCLC: non-small cell lung cancer; MCP: Manual coaching program; ACP: automated coaching program;VATS: video-assisted thoracic surgery; RATS: Robotic-assisted thoracic surgery; carbo-pem: Carboplatine-Pemetrexed-; cis-vino: Cisplatine-Vinorelbine; gem-cis: Gemcitabine_Cisplatine; Timing since surgery = number of days between randomization and surgery date.

Table S4. Actual Usage of the ACP and MCP Interventions.

		**MCP group**	**ACP group**	**P-value**
Project-tailored questionnaire	Answers	N (%)	N (%)	
1. How many times a day did you look at the application?	Never	0 (0)	0 (0)	0.76
	Once or twice a week	1 (11)	2 (20)	
	Sometimes, not every day	1 (11)	0 (0)	
	Once or twice a day	3 (33)	5 (50)	
	Multiple times a day	4 (44)	3 (30)	
2. How many times a day did you look at the activity tracker?	Never	0 (0)	1 (10)	0.33
	Once or twice a week	0 (0)	0 (0)	
	Sometimes, not every day	0 (0)	0 (0)	
	Once or twice a day	2 (78)	0 (0)	
	Multiple times a day	7 (78)	9 (90)	
Fitbit data		Median (IQ1;IQ3)	Median (IQ1;IQ3)	
Fitbit wear time		7 (7;7)	7 (6.87;7)	0.50

NoteI. ACP: Automated Coaching Program; MCP: Manual Coaching Program. Mann-Whitney U and Fisher's exact tests were performed.

Table S5. Feasibility of the ACP and MCP Intervention.

**Intervention feasibility**		**MCP group**	**ACP group**	**P-value**
Project-tailored questionnaire				
Questions	Answers	N (%)	N (%)	
1. How easy was it to use the smartphone application?	Very easy	0 (0)	5 (50)	0.04
	Easy	7 (78)	4 (40)	
	Not easy, but manageable	1 (11)	1 (10)	
	Difficult	1 (11)	0 (0)	
	Very difficult	0 (0)	0 (0)	
2. How easy was it to learn to use the application?	No/limited instructions	0 (0)	0 (0)	0.18
	Manageable after spending a lot of time/effort	0 (0)	0 (0)	
	Manageable after spending some time	4 (4)	1 (10)	
	Easy to learn to use	1 (11)	4 (40)	
	Easy to use immediately	4 (44)	5 (50)	
Data Interview				
Questions	Technical aspect	Explanation	Explanation	
1. How did you experience the technical aspects of this intervention?	Smartphone	11% needed time to adapt	30% minor difficulties manageable with some help via telephone contact with coach<br><br>30% sometimes difficulties with data synchronizing	
	Activity tracker	20% problems reading data on screen<br><br>10% watch strap broke during intervention	20% problems reading data onscreen<br><br>10% forgot charging batteries sometimes	
2. What do you think of the time spent on the intervention?		100% not time consuming	100% not time consuming	
**Coach feasibility**		(min/ID)	(min/ID)	
Contact time		54 ± 15	25 ± 14	0.0003

Note. ACP: Automated Coaching Program; MCP: Manual Coaching Program; M: Mean; SD: Standard Deviation; min: minutes. Mann-Whitney U and Fisher's exact tests were performed.
